# An interdisciplinary clinical practice model for the management of low-back pain in primary care: the CLIP project

**DOI:** 10.1186/1471-2474-9-54

**Published:** 2008-04-21

**Authors:** Stéphane Poitras, Michel Rossignol, Clermont Dionne, Michel Tousignant, Manon Truchon, Bertrand Arsenault, Pierre Allard, Manon Coté, Alain Neveu

**Affiliations:** 1 Montreal Department of Public Health, McGill University, Montreal, Canada; 2 Department of Rehabilitation, Laval University, Quebec City, Canada; 3 Department of Rehabilitation, Sherbrooke University, Sherbrooke, Canada; 4 Department of Industrial Relations, Laval University, Quebec City, Canada; 5 School of Rehabilitation, University of Montreal, Montreal, Canada; 6 Sir Mortimer B Davis Jewish General Hospital, Montreal, Canada; 7 Jewish Rehabilitation Hospital, Montreal, Canada; 8 Constance Lethbridge Rehabilitation Centre, Montreal, Canada

## Abstract

**Background:**

Low-back pain is responsible for significant disability and costs in industrialized countries. Only a minority of subjects suffering from low-back pain will develop persistent disability. However, this minority is responsible for the majority of costs and has the poorest health outcomes. The objective of the Clinic on Low-back pain in Interdisciplinary Practice (CLIP) project was to develop a primary care interdisciplinary practice model for the clinical management of low-back pain and the prevention of persistent disability.

**Methods:**

Using previously published guidelines, systematic reviews and meta-analyses, a clinical management model for low-back pain was developed by the project team. A structured process facilitating discussions on this model among researchers, stakeholders and clinicians was created. The model was revised following these exchanges, without deviating from the evidence.

**Results:**

A model consisting of nine elements on clinical management of low-back pain and prevention of persistent disability was developed. The model's two core elements for the prevention of persistent disability are the following: 1) the evaluation of the prognosis at the fourth week of disability, and of key modifiable barriers to return to usual activities if the prognosis is unfavourable; 2) the evaluation of the patient's perceived disability every four weeks, with the evaluation and management of barriers to return to usual activities if perceived disability has not sufficiently improved.

**Conclusion:**

A primary care interdisciplinary model aimed at improving quality and continuity of care for patients with low-back pain was developed. The effectiveness, efficiency and applicability of the CLIP model in preventing persistent disability in patients suffering from low-back pain should be assessed.

## Background

Low-back pain (LBP), e.g. pain in the lumbosacral area of the spine, is one of the most prevalent health problems in industrialized countries, engendering significant disability and costs. Back pain will generally resolve itself in the short term, with only a minority of patients developing persistent disability[1], disability defined as a reduction in an individual's capacity to perform usual activities, including work. However, this minority of patients is responsible for the majority of costs and has the poorest health outcomes. There is also scientific consensus that predictors of persistent disability are more psychosocial than biomedical in nature[2]. Thus, a shift of clinical focus from pathophysiology to the prevention of persistent disability is needed in primary care clinicians involved in LBP management[3]. Interdisciplinarity has also been proposed as a way to effectively address the multidimensional aspects of persistent disability related to LBP[4].

Several guidelines have been published on the clinical management of LBP[5], but they tend to devote a great deal of space to the efficacy of individual therapeutic interventions in LBP in general, and not on the prevention of persistent disability and process of care. A workshop held at the Fifth International Forum on Low Back Pain in Primary Care in Montreal (Canada) in May 2002 highlighted the fact that there were variations and lack of coordination among primary care clinicians in the management of LBP, resulting in inefficient care. It concluded that practice guidelines jointly developed by researchers and clinicians from various disciplines were needed[6]. The previous elements and the lack of guidelines in LBP management in the province of Quebec (Canada) triggered a movement to bring the different stakeholders in the province to work together on the elaboration of an interdisciplinary primary care LBP model aimed at the prevention of persistent disability. This process was a direct result of the previously mentioned workshop.

The objective of this project was to develop an interdisciplinary model aimed at the clinical management of adult LBP in primary care, with the aim of preventing persistent disability. The model address the following question: What actions should be taken by primary care providers when an adult presents with LBP in the acute, subacute or persistent stages of the condition, in order to prevent or manage persistent disability? The model was to contribute to better quality and continuity of care for adult patients with LBP.

## Methods

The Clinic on Low-back pain in Interdisciplinary Practice (CLIP) initiative was created and led by a project team (manuscript authors) representing research, academic and clinical experiences: one occupational health physician researcher, two physiotherapist researchers, one occupational therapist researcher, one psychologist researcher, two family physicians, and one physiotherapist clinician. A set of guidelines was chosen by the project team as a base. The Royal College of General Practitioners (RCGP) LBP guidelines[7] published in 2001 was chosen because it is a primary care multidisciplinary guideline of high quality[5]. The RCGP guidelines were updated by asking all project team members to independently search Medline, Embase and Cochrane libraries using the following strategy: "Back pain" in the title or subject heading; published in English; limited to systematic reviews, meta-analyses or RCTs; and published between 2000 and September 2005. They were asked to only select evidence related to primary care conservative management of adult LBP.

Numerous meetings were used by the project team to elaborate the model. During these meetings, project team members brought their findings. Criteria from RCGP guidelines were used to assess the quality of RCTs, with high quality studies having at least 10 patients in each group, and using patient centered validated outcomes (criteria from RCGP guidelines)[7]. The Oxman checklist was used to assess the quality of systematic reviews and meta-analyses[8]. Through discussions and consensus, the project team members identified shifts in evidence since the 2001 RCGP guidelines, and key evidence that would have the greatest impact on the prevention of persistent disability related to LBP. The project team designed the model in two sections composed of specific elements: 1) evaluation of LBP; 2) therapeutic approach of LBP. Using this evidence, project team members jointly elaborated elements of the model on a maximum of one page, including a clinical management statement, brief description of scientific evidence in support of the statement, an interpretation in terms of best practice options and a short list of references selected for educational purposes. Examples of tools to apply the model, such as questionnaires, were also provided. Using e-mail, several iterations of the model was circulated among project team members until there was consensus.

An interdisciplinarity community of practice was created by the project team. They approached 10 key stakeholders from the family physician, physiotherapy and occupational therapy licensing boards and clinician associations. These three groups of professionals provide the vast majority of primary care treatments to workers suffering from LBP in the province. Stakeholders were asked to identify and invite members that would be interested in LBP management. A group of 136 clinicians was assembled and asked to comment the feasibility of the model through online discussion forums and commentary forms. A seven member independent scientific committee, composed of researchers from five universities and of various disciplines (orthopedics, occupational therapy, physiotherapy, epidemiology, rheumatology and anthropology), provided comments on the validity of the model. Taking into account comments, the project team decided by consensus if and how the model should be revised, without deviating from the evidence. The final model was summarized in the form of a clinical algorithm.

## Results: Model elements

### 1. Assessing the patient

#### 1.1. In order to detect serious problems requiring immediate or specialized treatment, the clinical examination should triage patients according to the three types of low back pain: non-specific, with neurological involvement, with serious pathology (red flags)

Table 1 describes the characteristics of the three types of low back pain. The most common recommendation published throughout the world in clinical practice guidelines concerns initial patient triage[9]. The main sought after goal is the identification of red flags (category "C") requiring immediate medical or surgical attention[10]. In general, patients with neurological signs and symptoms (category "B") progress statistically twice as slow as patients with simple back pain (category "A")[11,12].

**Table 1:** Three types of low-back pain to be used in patient triage

**A) Non-specific back pain**
General characteristics:
- Lumbar or lumbosacral pain with no neurological involvement
- "Mechanical" pain, varying over time and with physical activity
- Patient's general health is good
**B) Back pain with neurological involvement**
The patient should have one or more symptoms **and**signs indicating possible neurological involvement.
**Symptoms**
- Pain radiating below the knee, which is as intense or more intense than the back pain
- Pain often radiating to the foot or toes
- Numbness or paresthesia in the painful area
**Signs**
- Positive sign for radicular irritation as tested, for example, by straight leg raising
- Motor, sensitivity or reflex signs supporting nerve root involvement.

**C) Back pain with suspected serious spinal pathology (red flags)**
General characteristics:
- Violent trauma (such as a fall from height or an automobile accident)
- Constant, progressive, non-mechanical pain
- Thoracic or abdominal pain
- Pain at night that is not eased by a prone position
- History of or suspected cancer, HIV or other pathologies that can cause back pain
- Chronic corticosteroid consumption
- Unexplained weight loss, chills or fever
- Significant and persistent limitation of lumbar flexion
- Loss of feeling in the perineum (saddle anesthesia), recent onset of urinary incontinence
The risk of a serious condition may be higher in those under 20 or over 55 years of age. Particular attention should be paid to the previously mentioned signs and symptoms in patients in these age groups.

#### Interpretation

Red flags are warning signs that should lead the clinician to investigate for a serious pathology in need of immediate diagnosis (category "C"). These are mainly lumbar complications from a serious trauma or a disease such as cancer. In practice, such complications are rare but systematic questioning and examination is required in order to detect them[10]. Neurological signs and symptoms in the patient with back pain with no red flags (category "B") often resolve themselves without recourse to surgery[10]. A referral for a specialized consultation should not be required until the clinician has observed a functional deficit that is persistent or deteriorating[10] after four weeks. Hence, aside from observing the progression of neurological signs and symptoms, management of these patients is identical to that for simple pack pain (category "A"). Diagnostic triage can be repeated when needed according to progression. Diagnostic triage of low back pain is useful in screening for red flags and weighing the urgency of medico-surgical treatment. It does not exclude the use of validated sub-categories to guide treatment choices and adjustments. Although commonly recommended, there is no direct evidence that triaging positively impacts patient outcomes.

#### 1.2. Radiographic, MRI or CT scan examinations are rarely indicated for patients with non-specific back pain

In patients suffering from simple low back pain, X-ray, CT scan or MRI results are not associated with the symptoms described by the patient or his perceived disability.

A review on the relationship between simple back pain and X-ray results concluded that there is no evidence of a causal relationship between X-ray findings, particularly degenerative changes, and simple back pain[13]. For the two other types of back pain, particularly in patients over 55 years of age, a literature review concluded that simple X-ray were sufficient to exclude spinal pathology[14]. Specialized imaging tests (such as CT scan and MRI) should be reserved for cases in which surgery is being considered or where there is a strong suspicion of systemic disease.

#### Interpretation

When patient history and physical examination reveal no red flags, a reliable clinical diagnosis can be made without recourse to medical imaging techniques[14]. When specialized diagnostic imaging examinations are performed e.g. a scan or MRI, results should always be interpreted in light of the clinical findings. Unnecessary use of these highly sensitive examinations will produce numerous false positive results[13], which can create a labelling effect for the clinician and his patient that can in itself contribute to a less favourable prognosis[15].

#### 1.3. The clinician should assess the patient's perceived disability and the probability of a return to usual activities, either in the fourth week if back pain related disability persists, or at the first consultation if the patient has a history of long lasting disability due to back pain

The probability of return to work decreases with the length of disability due to low back pain, creating three stages: Acute (less than 4 weeks of disability); subacute (4 to 12 weeks of disability); persistent (more than 12 weeks of disability)[16]. The study of the relationship between a longer absence from work and a weaker probability of return to usual activities has shown reproducible results. A review shows that the progression of prognosis in relation to the duration of back pain is confirmed not only for return to work but also for level of perceived disability[1]. The assessment of perceived disability to determine the impact of low back pain on the patient's health is one of the recommendations most frequently found in practice guidelines[9].

#### Interpretation

The classification of low back pain into stages permits the identification of the turning points (acute, subacute and persistent) at which the clinician should adapt the treatment on the basis of a deteriorating prognosis[1]. This adjustment is determined in part by the prediction of persistent disability[17]). Examples of prediction rules for persistent disability are the Symptom Checklist Back Pain Prediction Model (SCL BPPM) questionnaire[18] or the "Recherche sur les Affections Musculo-Squelettiques" (RAMS) questionnaire[19] The SCL BPPM can be used for the general population, while the RAMS can be used for workers. When the rule predicts persistent disability (SCL BPPM: moderate or elevated risk of disability; RAMS: partial success or failure to return to work), the clinician should intensify the search for the barriers preventing the return to usual activities or refer the patient to a clinician capable of identifying such barriers[17]. Adjustment of management also depends on the assessment of the patient's perceived disability using a standardized questionnaire. Examples of tools that can be used for this assessment are the "Quebec back pain disability scale"[20], the Roland-Morris Disability Questionnaire[21] or the Oswestry Disability Questionnaire[22].

#### 1.4. When the probability of returning to usual activities is deemed to be low (Principle 1.3), the clinician should seek to identify the barriers preventing the return to usual activities

There is a high level of evidence supporting the influence of certain clinical, psychosocial and work-related factors in the probability of returning to usual activities[2]. In order to reduce their impact, these factors or barriers should be identified[17]. The identification of the barriers preventing the return to usual activities is one of the most commonly recurring recommendations in clinical practice guidelines published internationally[23].

#### Interpretation

As mentioned in Principle 1.3, where the likelihood of returning to daily activities is deemed to be low, the clinician should intensify his efforts to identify barriers preventing the return to usual activities. By identifying these barriers, the clinician can adapt treatment or quickly refer the patient to other resources if necessary to avoid chronicity[17]. A review[2] identified the barriers having a major impact on the ability to return to usual activities. These barriers appear to be interrelated, that is, when improvement is obtained in one area it results in improvement in the others[24]. The following describes the key barriers that should be assessed as well as examples of tools that can be used in their assessment:

• Intensity of pain (visual analogue scale)

• Perceived disability (Quebec Back Pain Disability Scale[20] or Roland-Morris Disability Questionnaire[21] or Oswestry Disability Questionnaire[22])

• Symptoms (with no signs) of radiating pain below the knee (Clinical consultation)

• Fears and beliefs (Tampa Scale for Kinesiophobia)[25]

• Patient projection regarding return to work (3-month projection question in the Fear-Avoidance Beliefs Questionnaire)[26]

• Catastrophizing (Pain Catastrophizing Scale)[27]

• Absence from any type of work (Employment status)

#### 1.5. If the patient's perceived disability improves little or not at all in the 4 weeks following assessment of this perception, the clinician should reassess the barriers preventing the return to usual activities and revise management

Patient perceived disability has been demonstrated in the literature to be related to the barriers influencing the return to usual activities mentioned in Principle 1.4[2,17]. Lack of or slow progression of this perception can indicate that barriers preventing the return to usual activities are present and should be identified and managed[17].

#### Interpretation

A perceived disability questionnaire can be used at four week intervals. The score obtained with this assessment should improve by a certain amount (Quebec Back Pain Disability Scale: at least 15 points; Oswestry Disability Questionnaire: at least 10 points; both out of 100[28]) over a period of four weeks. Little or no improvement is an indication that the clinician should look for barriers preventing the return to usual activities[17]. Moreover, where the progress of the patient's back pain and perceived disability is slow but regular, a referral to a rehabilitation clinic can be indicated where a program aimed at the return to usual activities will be undertaken.

### 2. Therapeutic approach

#### 2.1 Reassure the patient with back pain by (1) providing essential, coherent, accessible and valid information about his condition and (2) correcting beliefs

Interest in the importance of the type of information given to patients with low back pain at the first consultation and thereafter is relatively recent. Two corroborating studies on the subject have shown that essential, coherent and accessible information can have a positive impact on the patient's recovery [29,30]. Essential information consists of a limited number of clear messages (three to five). Coherent information is the clinician's verbal information accompanied by a written document containing the same information. Accessible information is that which is adapted to the patient and the patient's health.

#### Interpretation

Information given to the patient with low back pain is important because it allows the patient to understand what is at stake therapeutically and become involved in his functional recovery. However, information can be a double-edged sword since contradictory or poor quality information can work against the patient's wellbeing and slow down the return to usual activities[31]. Regarding the available information on low back pain, two studies, three years apart highlighted the poor quality of that information available in 90% of English language web sites[32,33]. Today, patients have access to tens of thousands of web sites on back pain alone increasing the importance of the clinician's role in providing information particularly in correcting beliefs and perceptions. Several tools have been developed to provide validated information to the patient with back pain. "The Back Book"[34] is an example of works that have contributed to rendering the information coherent among clinicians and improving patient access to quality information, while respecting the spirit of clinical practice guidelines. Among the key messages contained in the Back Book to convey to the patients, the following are noted:

• Reassure the patient about the generally positive prognosis of back pain;

• Reassure the patient that serious spinal problems are rare and that the signs (red flags) for such problems are not present;

• Reassure the patient regarding returning to or continuing usual activities, including work, even in the presence of symptoms;

• Avoid labelling the patient by putting an exaggerated emphasis on a specific spinal problem and its impact.

#### 2.2 The clinician should encourage and guide the patient to continue or to resume usual activities

Evidence supported by high quality studies show the superior advantage of encouraging activity to prescribing bed rest[35]. Although superficially dissimilar, the convergent results of these studies illustrate varying aspects of the principle of remaining active while never contradicting it. To remain as active as possible is the most widely respected clinical and scientific recommendation in the world today [9].

#### Interpretation

The patient advised to continue or to resume daily activities including work and to avoid bed rest as much as possible recovers more quickly than the patient who is advised to be guided by pain in resuming activity[36]. Although throughout the world this recommendation is the most widely found in clinical practice guidelines, a review noted that, in general, practice guidelines lack an explanation of how the clinician might meet this therapeutic objective with the patient[23]. Another criticism of this recommendation has been a lack of sensitivity to the individual context of the patient, increasing the difficulty of the clinician's job. Consistency among messages delivered to the patient by clinicians from one visit to the next might well be the most important parameter in implementing Principle 2.2. Encouragement to remain active is a recommendation that is subordinate to the information provided to the patient and to the correction of beliefs (Principle 2.1). Examples of tools for the evaluation and management of barriers are available to guide the return to work[37].

#### 2.3 The clinician should give priority to treatments of proven efficacy

Numerous therapeutic interventions have been proposed for the treatment of low back pain. In recent years considerable research has been devoted to the rigorous evaluation of the most common therapeutic interventions. The syntheses of these Cochrane type studies or the most up to date meta-analyses were compiled to create tables 2, 3 and 4 classifying therapeutic modalities according to their level of scientific evidence in the stages of low back pain. Strength of evidence is based on the following criteria[7]:

**Table 2:** Therapeutic interventions for acute low-back pain (0–4 weeks)

**Grade of scientific evidence**			
**Strong**	**Moderate**	**Poor**	**Lack of evidence**
	
**Can be recommended**			

**NSAIDs **[51-54]	**Vertebral manipulations**	**Steroid epidural infiltration for radicular pain **[53]	**Physical agents (ice, heat, diathermy, ultrasounds) **[58,59]
- Efficacy to ↓ pain = acetaminophen for all NSAIDs	- Efficacy > placebo [53]	- Efficacy > placebo or bed rest	
	- Efficacy > mobilisation for short term pain reduction [55]		
	- Efficacy = conservative treatment [56,57]		
**Muscle relaxants **[53,60]	**Exercises for disc herniation **[42]	**Analgesics **[52–54]	**Antidepressants **[52,53,61]
-Efficacy of non-benzodiazepines > benzodiazepines; both with potential harm	- Efficacy of extension > flexion	- Non-opioids as efficacious as NSAIDs for pain relief	
		- Opioids: weak evidence of superiority to non-opioids	
**Combination relaxants + NSAIDs or analgesics **[60]		**Lumbar support **[53]	**Facet infiltrations **[53]
- Efficacy > placebo		- Weak efficacy compared to no treatment	
		- Efficacy unknown compared to conventional therapies	
		- No efficacy for prevention	
**Advice to remain active **[36,59]		**Acupuncture **[62,63]	**Steroid epidural infiltration for non-radicular pain **[53]
- Efficacy > conventional medical treatment		**Steroid drugs **[53]	**Back schools **[65]
		**McKenzie approach **[66]	**Massage **[57,67]
	
**Cannot be recommended**			
	
**Bed rest **[53,64]	**Exercises in flexion **[42]	**TENS **[53,58,68]	
		- Weak efficacy compared to other treatments	
		- No efficacy in meta-analysis	
**Strengthening exercises **[42]			
**Specific exercises **[42]			
**Mechanical tractions **[58,68,69]			
**Exercises in extension **[42]			

NSAID: non-steroidal anti-inflammatory drugs

TENS: transcutaneous electrical nerve stimulation

**Table 3:** Therapeutic interventions for subacute low-back pain (4–12 weeks)

**Grade of scientific evidence**			
**Strong**	**Moderate**	**Poor**	**Lack of evidence**
	
**Can be recommended**			

**Advice to remain active **[36,53,64]	**McKenzie approach **[66]	**Acupuncture **[62]	**Lumbar support **[59,70]
- Graded activity + behavioral intervention = ↓ absence from work and ↓ risk of chronicity			

**Exercises **[42,68]	**Multidisciplinary program **[39,59]	**Vertebral manipulations **[56,57]	**TENS **[68]
- no superiority of one type compared to another	- efficacious if intensive, includes return to work component with visit of workplace.	- Efficacy > placebo [53]	
		- Efficacy > mobilisation to reduce short term pain [55]	
		- As efficacious as other conservative treatments	
		**Massage **[67]	**Radiofrequency denervation **[71]
		- Efficacy > no treatment	
		- Better efficacy if combined to exercises and education	
		**Behavioral therapy **[59]	**Physical agents (ice, heat, diathermy, ultrasounds) **[53]
		- Efficacy on pain and functional limitations > traditional care	
		**NSAIDs **[51]	**Steroid epidural infiltration **[53]
		- Efficacy to ↓ pain = acetaminophen for all NSAIDs	
		**Analgesics **[52–54]	**Infiltration of trigger points **[53,72]
		- Non-opioids as efficacious as NSAIDs for pain relief	
		- Opioids: weak evidence of superiority to non-opioids	
	
**Cannot be recommended**			
	
		**Bed rest **[64]	
		**Mechanical tractions **[68,69]	

NSAID: non-steroidal anti-inflammatory drugs

TENS: transcutaneous electrical nerve stimulation

**Table 4:** Therapeutic interventions for persistent low-back pain (12 weeks +)

**Grade of scientific evidence**			
**Strong**	**Moderate**	**Poor**	**Lack of evidence**
	
**Can be recommended**			

**Multidisciplinary program **[39,73]	**Back school **[65,74]	**Massage **[57,67,74,75]	**Lumbar support **[70,74,75]
- Efficacious if intensive, includes return to work component with visit of workplace.	- Efficacy if short term and on workplace premises	- Efficacy > no treatment	
		- Better efficacy if combined to exercises and education	
**Behavioral therapy **[41,74]		**NSAIDs **[51,52,74]	**Prolotherapy injection **[76]
- Efficacy > no treatment or waiting list if includes cognitive approach and relaxation		- Efficacy to ↓ pain = acetaminophen for all NSAIDs	
**Exercises **[42,58,74]		**Vertebral manipulations **[55,56,75]	**Neuroreflexotherapy **[78]
- No superiority of one type compared to another			
- Better if individualised			
		**McKenzie approach **[66]	
		**Muscle relaxants **[52,61]	
		- Evidence weaker than in acute phase	
		- Advantage over benzodiazepines	
		**Antidepressants **[52,61]	
		- Efficacy > placebo	
		- Advantage for tricyclic and tetracyclic	
		**Acupuncture **[62,63]	
		- Efficacy on pain and functional status	
		- Efficacy = other treatments	
		**Steroid epidural infiltration **[72,74]	
		**Infiltration of trigger points **[72,74]	
		**Radiofrequency denervation **[71,79]	
	
**Cannot be recommended**			
	
**Bed rest **[58,64,68,74]	**Injection therapy **[72,74]	**Therapeutic ultrasounds **[68,75]	
**Mechanical tractions **[58,74,75]	**TENS **[58,75,77]		

NSAID: non-steroidal anti-inflammatory drugs

TENS: trans-cutaneous electrical nerve stimulation

• Strong: consistent findings in several high quality studies;

• Moderate: consistent findings in lesser quality studies, particularly with small numbers of subjects;

• Poor: Based on the results of only one study or inconsistent findings in several studies

• Lack of evidence: Based on studies with no comparison group, on theoretical considerations or on expert consensus.

#### Interpretation

Each of the modalities is qualified as "recommendable", "not recommendable" or "unknown efficacy". Because the design of the tables requires some interpretation of the source documents, it is necessary to refer directly to them to understand the meaning and impact of these recommendations. Clinical application methods can vary considerably among the studies and the meaning of the conclusions can differ according to clinical context.

In addition, there are many treatments for which no studies exist and no recommendation can be made. Further studies are necessary before it is possible to rate their efficacy. The lack of scientific evidence does not in itself discredit a treatment

#### 2.4 When individual or environmental barriers to the return to usual activities are identified after the acute phase of low back pain, the clinician should reorient treatment towards minimizing those barriers

As mentioned in Principle 1.3, the possibility of returning to usual activities diminishes significantly with the approach of persistent low back pain. In addition, the risk of persisting symptoms is greater. Evidence related to the treatment of sub-acute and persistent low back pain is concerned primarily with communication and the multidimensional nature of the barriers preventing the return to usual activities[38]. With regards to communication, the primary clinical concerns rests on the sharing of common information among the caregivers involved in treatment[6]. Regarding the multidimensional nature of barriers present in the patient with low back pain, a review emphasized the importance of acting on both the individual (physical and psychological) and environmental (social and work-related) levels[39].

When the patient does not return to all or some activity after 12 weeks of back pain, the possibility of returning to usual activities decreases significantly and the risk of persisting symptoms increases[6]. The literature indicates that the barriers to returning to activity for the persistent low back pain sufferer are not only physical but are also and foremost biopsychosocial, including the patient's environment[2]. The clinician should identify the limiting barriers and attempt, with the patient, to understand why and how these barriers interact in limiting return to usual activities[38].

#### Interpretation

Care should be oriented towards the identification and management of individual and environmental barriers preventing the return to usual activities (see Principle 1.4) and on decreasing symptom-based treatment. This change can be done by encouraging patient participation in his management of low back pain and by involving the stakeholders who can contribute to diminishing the barriers[38].

Once persistent disability is present, the multiplicity and entrenchment of individual and environmental barriers results in a handicap that keeps the individual from returning to usual activities. A review identified most of the barriers that limit the return to usual activities, including work, in the presence of persistent low back pain[40]. They reiterate most of the barriers discussed in Principle 1.4 but in the context of persistent back pain. Only those barriers that could potentially be modified by the clinician should be addressed (age, for example, cannot be modified). The clinician should systematically identify these barriers in order to understand their impact on the patient's handicap and to account for them in the treatment plan[40]. A treatment can act directly or indirectly on barriers to returning to usual activities. For example, behavioural therapy or generic exercises can both have an impact on patient fears and beliefs, the former directly[41] and the latter indirectly[42]. Hence, the clinician's objective is to choose the interventions that will best act to change the barriers identified.

Although standardized tools to assess the extent of barriers to return to usual activities are proposed, precise interventions to manage these barriers are not available in the literature. It is not known if these barriers are modifiable by a clinician, or how they should be managed. Also, it is not known if managing these barriers can actually prevent persistent disability. This reflects the current relative lack of knowledge in the literature in this area needing further research[43].

When the clinician feels that help is needed to facilitate the return to usual activities for a patient suffering from persistent low back pain, he can refer the patient to specialized resources available in his area. The primary care clinician remains a resource for the patient throughout the rehabilitation process and during subsequent low back pain episodes.

### 3 Clinical algorithm

**Figure 1:**
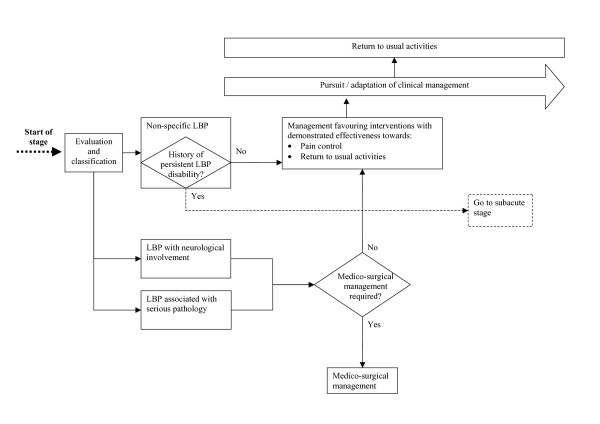
Clinical algorithm for acute low-back pain.

**Figure 2:**
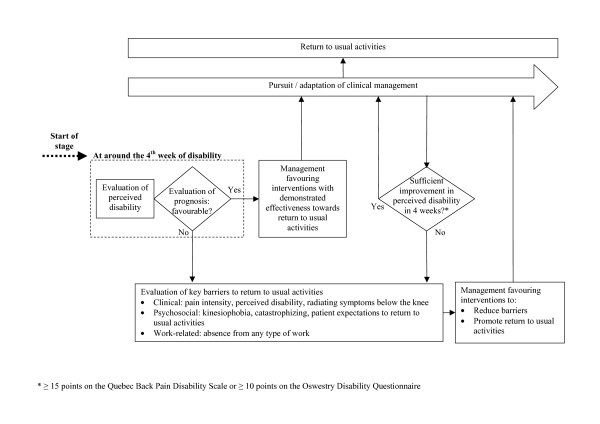
Clinical algorithm for subacute low-back pain.

**Figure 3:**
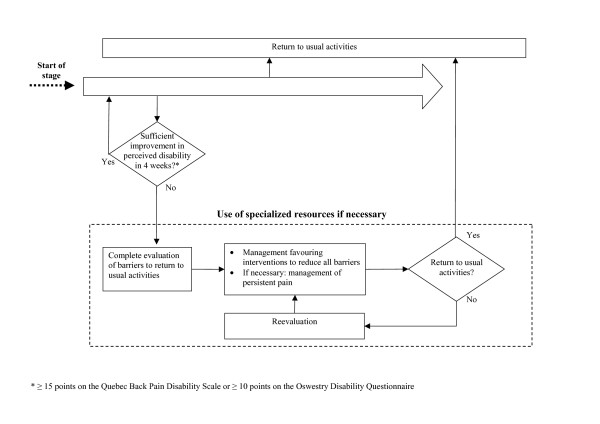
Clinical algorithm for persistent low-back pain.

Figures 1 to 3 show the model's algorithm through different stages of LBP: acute (less than 4 weeks), subacute (4 to 12 weeks) or persistent (more than 12 weeks). Figure 2 shows the section of the algorithm for subacute LBP, where are situated three central elements aimed at the prevention of persistent disability. The first is the evaluation of the prognosis at the fourth week of disability, and of key modifiable barriers to return to usual activities if the prognosis is unfavourable. The second is the evaluation of the patient's perceived disability every four weeks, with the evaluation of barriers to return to usual activities if perceived disability has not sufficiently improved.

## Discussion

This process had the objective of developing a primary care interdisciplinary model aimed at preventing persistent disability related to LBP. The CLIP model was designed for family physicians, physiotherapists and occupational therapists, irrespective of their specific expertise. It's goal was to promote the use of similar tools and common language in the management of LBP from a bio-psycho-social perspective. The model promoted common therapeutic goals in terms of returning the patients to their usual activities, periodic evaluation of perceived disability and re-orientation of care when limited results are achieved. The shift of focus from specific therapeutic interventions to process of care has two important implications for clinical practice: 1) evaluate the patient's perceived disability with a validated instrument; 2) identify barriers to return to activity and to orient the patient's management accordingly. The CLIP model can be used in interdisciplinary training sessions, with several types of health professionals present at the same time.

This model was developed through a rigorous process of literature search and synthesis, and interdisciplinary exchanges among researchers and health professionals. The interpretation of the evidence on the efficacy of therapeutic interventions provided in the summary tables was consistent with previously published guidelines[5,23]. However, this efficacy is evaluated for each individual treatment, which does not reflect everyday clinical practice combining different types of interventions pursuing different objectives. Future trials assessing the efficacy of representative clinician practices are needed. This model emphasises the assessment and management of barriers related to return to activity, because of their strong and consistent predictive relationship to persistent disability. However, the effectiveness and efficiency of this model to prevent persistent disability in patients suffering from LBP are not known and should be assessed. Research in ways to effectively address barriers to return to activity is needed.

This model does not take into account the time, resources and costs needed to use it. Resistance in use should be expected, since this model requires a shift from a pathophysiological to a biopsychosocial model of disease management. Difficulties in using such evidence have been previously demonstrated [44-49]. In order to evaluate the model's feasibility, it should be piloted among end users working in various in various clinical, organizational and geographical settings, and among patients with varying level of disability. Taking into account the identified barriers and facilitators in use, adapted versions of the model should be elaborated[50]. The process used for the development of the model remains in place and will be used for further interdisciplinary exchanges and integration of new evidence. It is planned that the model will be updated in the end of 2008. It is hoped that shared use of this model by primary care health professionals will prevent persistent disability and its consequences in persons suffering from LBP.

## Conclusion

A model for the clinical management of low-back pain and prevention of persistent disability was developed. The following five elements were elaborated for the evaluation of LBP: 1) In order to detect serious problems requiring immediate or specialized treatment, the clinical examination should triage patients according to the three types of low back pain: non-specific, with neurological involvement, with serious pathology (red flags); 2) Radiographic, MRI or CT scan examinations are rarely indicated for patients with non-specific back pain; 3) The clinician should assess the patient's perceived disability and the probability of a return to usual activities, either in the fourth week if back pain related disability persists, or at the first consultation if the patient has a history of long lasting disability due to back pain; 4) When the probability of returning to usual activities is deemed to be low, the clinician should seek to identify the barriers preventing the return to usual activities; 5) If the patient's perceived disability improves little or not at all in the 4 weeks following assessment of this perception, the clinician should reassess the barriers preventing the return to usual activities and revise management. The following four elements were elaborated for the management of LBP: 1) Reassure the patient with back pain by providing essential, coherent, accessible and valid information about his condition and correcting beliefs; 2) The clinician should encourage and guide the patient to continue or to resume usual activities; 3) The clinician should give priority to treatments of proven efficacy; 4) When individual or environmental barriers to the return to usual activities are identified after the acute phase of low back pain, the clinician should reorient treatment towards minimizing those barriers.

## Competing interests

The authors declares that they have no competing interests.

## Authors' contributions

All authors participated in conception and design of the study, acquisition of data, interpretation of data, and revision of the manuscript. All authors read and approved the final manuscript. SP and MR additionally analyzed the data and drafted the manuscript.

## Pre-publication history

The pre-publication history for this paper can be accessed here:


